# Foliar application of humic acid improves growth, yield, and nutritional quality of mungbean (*Vigna radiata L.*) genotypes under newly reclaimed soil conditions

**DOI:** 10.1038/s41598-026-47151-z

**Published:** 2026-04-17

**Authors:** D. M. Sabra, Elham A. Badr, Magda H. Mohamed, Gehan A. Amin

**Affiliations:** Field Crops Research Department, Agricultural and Biological Research Institute, National Research Centre, 33 El Bohouth St., P.O. 12622, Giza, Egypt

**Keywords:** Mungbean, Humic acid, Foliar application, Genotypes, Amino acids, Productivity, Physiology, Plant sciences

## Abstract

Egypt faces a seasonal shortage of edible pulses during the summer months; therefore, introducing high-yielding legume crops such as mungbean (***Vigna radiata L.)*** may contribute to improving pulse production. Two field experiments were conducted at the Nubaria Research Station, Egypt, during the summer seasons of 2022/2023 and 2023/2024 to evaluate the effect of foliar application of humic acid (HA) at three levels (0, 10, and 20 mL L^− 1^) on productivity and seed quality of five mungbean genotypes (Kawmy^− 1^, King, AVM125, VC1354, and VC3896). The results indicated that foliar application of HA significantly improved growth attributes, yield components, and nutritional quality compared with untreated plants. The application of HA at 20 mL L^− 1^ generally resulted in the highest values of vegetative growth and yield parameters. Significant genotypic variation was observed, where King and VC3896 exhibited superior performance, while AVM125 showed relatively lower growth response. The interaction between genotype and HA level significantly affected yield traits. The combination of King genotype with HA at 10 mL L^− 1^ recorded the highest seed yield (3.85 t ha^− 1^) and protein yield (0.24 t ha^− 1^), whereas Kawmy-1 with HA at 20 mL L^− 1^ produced the highest biological and straw yields. Foliar application of HA enhanced nutrient uptake and carbohydrate accumulation, which was reflected in improved seed quality and amino acid composition, particularly lysine, methionine, leucine, and valine.Under the conditions of this study, foliar application of humic acid may represent a promising agronomic approach for improving mungbean productivity and seed nutritional quality in newly reclaimed soils.

## Introduction

Mungbean (***Vigna radiata L***.) is an important summer legume crop belonging to the Fabaceae family, recognized for its high protein content (19–29%) and essential amino acids, particularly lysine, which make it a valuable component of human diets .Its seeds are also rich in carbohydrates, vitamins, and minerals such as calcium, zinc, and iron, while the plant residues can serve as livestock feed or green manure, improving soil fertility through nitrogen fixation^1^. The crop requires relatively low water input, offers favorable economic returns, and can be cultivated biannually, making it a suitable option for newly reclaimed lands with limited fertility. In Egypt, edible pulses are scarce during the summer months, and introducing mungbean into summer cropping systems could contribute to improving pulse production, dietary protein supply, and soil sustainability^2^. However, newly reclaimed soils often have low organic matter and poor nutrient availability, which limits crop productivity^3^. Therefore, strategies to enhance soil fertility and improve plant nutrient uptake are essential for successful mungbean cultivation in these conditions^4^. Different mungbean genotypes exhibit variation in growth, yield, nutrient use efficiency, and response to agronomic practices. Identifying genotypes that respond positively to foliar HA application can optimize productivity and seed quality, especially in marginal soils^5^. Previous studies have indicated that foliar HA can mitigate environmental stress, improve vegetative growth, and enhance reproductive performance in legumes. However, systematic evaluation of HA effects on multiple mungbean genotypes under newly reclaimed soil conditions remains limited^6^.

In newly reclaimed lands, soil organic matter is often low; therefore, exogenous application of humic substances may improve soil fertility and plant performance. Humic acids are classified as organic acids that are generated naturally from the decomposition of organic matter in the soil. In addition to, the use of humic acid is very important and has a positive effect under less than suboptimal conditions, Humic acid is a vital constituent and an intimate part of soil organic structure and contains 4% to 6% nitrogen, 51 to 57%, carbon and 0.2% to 1% phosphorus and other micronutrients^7^. These acids play a significant role in enhancing plant growth by facilitating the absorption of essential elements, thereby making them more accessible to plants. Additionally, humic acids positively influence plant hormones and their responsiveness by inhibiting the enzyme IAA oxidase, which results in increased levels of the IAA hormone that promotes plant growth. Furthermore, humic acids exhibit effects akin to auxins, which stimulate root development^8^. They contribute to the enhancement of the physical, chemical, and biological characteristics of soil, while also alleviating issues related to salinity and mitigating the adverse effects of water and soil conditions^9^ Moreover, humic acids help to reduce the impact of water stress on plants, thereby improving the root system’s capacity to absorb nutrients^10,11^ and^12^ demonstrated that how humic acid can mitigate water deficit stress and improve physiological responses in onion that supporting the relevance of HA in stress alleviation.

**Research gap and objective**: Despite evidence that HA improves plant growth and nutrient uptake, its impact on growth, yield, and amino acid composition of different mungbean genotypes under new land conditions has not been fully elucidated. This study aims to evaluate the effect of foliar application of humic acid at different concentrations on growth attributes, yield components, nutrient uptake, and amino acid composition of five mungbean genotypes. The findings are expected to identify optimal HA treatment and responsive genotypes, providing a sustainable approach to enhance mungbean productivity and seed quality in low-fertility soils.

## Materials and methods

### Ethical statement/plant material compliance

Plant Material and Ethical Compliance Statement: The mungbean (Vigna radiata L.) genotypes used in this study were obtained from authorized sources, including the Field Crops Research Department, National Research Centre (NRC), Egypt, and the Asian Vegetable Research and Development Center (AVRDC). No wild plant collection was conducted. All plant materials were cultivated genotypes commonly used in agricultural research, and therefore no specific collection permits were required. Plant identification was confirmed by National Research Centre (NRC).This study complies with all relevant institutional, national, and international guidelines. The research does not involve endangered or protected species and is consistent with the principles of the IUCN Policy Statement on Research Involving Species at Risk of Extinction and the Convention on International Trade in Endangered Species of Wild Fauna and Flora (CITES).

### Experimental design

Two field experiments were conducted in the summer (June 2022/2023 and 2023/2024) at the National Research Centre (NRC), Research and Production Station in Nubaria District, Al-Behaira Governorate, Egypt (latitude 30°30′1.4″ N, longitude 30°19′10.9″ E, 21 m above mean sea level) the experimental soil in (Table 1) were taken at a (0–30 cm depth) then was analyzed according to the method described by^13^, climatic data during the experimental period were obtained from El-Nubria Meteorological station (Table 2).

**Table 1:** Some physical and chemical characteristics of the experimental soil mechanical analysis.

Sand		Silt 20-0µ%	Clay < 2µ%	Soil texture
Course 2000-200µ%	Fine 200-20µ %			
45.93	37.27	11.93	4.78	Sandy

Chemical analysis											
pH 1:2.5	EC dSm^-1^	CaCo_3_	OM%	Soluble cations meq/l				Soluble anions meq/l			
				Na^+^	K^+^	ML^+^	Ca^++^	CO_3_^--^	HCO_3_^-^	Cl^-^	SO_4_^--^
7.37	1.15	4.93	0.15	0.53	0.15	0.72	1.07	0.02	1.42	0.52	0.76

Macro and micronutrients analysis						
Available nutrients						
Macro ppm		Micro ppm				
N	P	K	Zn	Fe	Mn	Cu
47.54	10.15	66.57	0.17	1.37	0.30	0.05

**Table 2:** Climatic data of the experimental site at El-Nubaria station (monthly average of two seasons 2022/2023 and 2023/2024).

Month	Max. Temp. °C	Min. Temp. °C	RH %	Wind Speed m/sec.	Rain mm
June	35.76	20.33	53.54	4.34	0.0
July	36.52	21.32	54.60	4.52	0.0
August	37.20	22.67	55.12	4.87	0.1
September	35.28	21.11	54.32	4.11	0.4

The experiment was arranged in a split-plot design with four replications. The main plots consisted of five mungbean genotypes: Kawmy-1 (local), King, AVM125, VC1354, and VC3896 (imported from the Asian Vegetable Research and Development Center, AVRDC). The subplots received foliar applications of humic acid (HA) liquid (5% w/v HA, 1% w/v fulvic acid, 2% w/v K2O, PH 8–9) at three levels: 0 mLL-1 (control), 10 mLL-1, and 20 mLL-1. Foliar spraying was applied twice, at 30 and 45 days after sowing, at a rate of 480 L ha-1. Each plot measured 10.5 m^2^, consisting of five rows, 3.5 m long, with 60 cm between rows. Seeds were sown manually in hills spaced 30 cm apart (2 seeds per hill on each side of the row) in mid-May of each season. Drip irrigation was applied immediately after sowing, and standard NPK fertilization was performed at rates of 80 kg N ha^− 1^ (ammonium nitrate, 33% N), 75 kg P_2_O_5_ ha^− 1^ (superphosphate, 15.5% P_2_O_5_), and 57 kg K_2_O ha^− 1^ (potassium sulfate, 48% K_2_O) (FAO, 2017). Conventional agronomic practices for mungbean cultivation, including weed control and pest management, were followed throughout the growing season.

### Growth characters

Ten randomly selected plants from each center plot were collected at harvest (90 DAS) in order to determine the yield components of the following characters:

Plant height (cm); number of branches, and Pods /plant; 100-seed weight (g) and fresh weight of plant (g).

### Biological yield

The whole yield of each plot (10.5 m^2^) was harvested for character to calculate: Seed yield (ton ha^− 1^); Straw yield (ton ha^− 1^); Biological yield (ton ha^− 1^); Protein Yield (ton ha^− 1^.) and Harvest index.

$${\text{Harvest index }}\left( \% \right){\text{ }}={\text{ }}\left[ {{\text{Grain yield }}\left( {{\text{ton h}}{{\mathrm{a}}^{ - {\mathrm{1}}}}} \right){\text{ }}/{\text{ Biological yield }}\left( {{\text{ton h}}{{\mathrm{a}}^{ - {\mathrm{1}}}}} \right)} \right]{\text{ }} \times {\text{ 1}}00.$$

### Chemical constituents

Protein %. Using the micro Kjeldahl equipment, the nitrogen and protein contents were measured in accordance with the methodology described by^14^. Estimation the crude protein concentration was determined according to^15^ by multiplying the nitrogen levels by 6.25. Total carbohydrates were determined according to^16^.

Amino acids %: Amino acid composition estimated of seeds by using Amino acid analyzer (Biochrom 30) according to^17^.

### Statistical analysis

All statistical assumptions were checked prior to analysis. The data were statistically analyzed using a split plot design according to^18^. Combined analysis of the two growing seasons was conducted for the data of the two seasons after tested the variances homogeneity of both seasons. Means were compared by using least significant difference (LSD) at 5% levels of probability. The Ward’s method is Correlation relationships and the clustering methodology and Euclidian distance is the measure of dissimilarity, and the application SAS v.9.1.3 was used to perform the cluster analysis according to^19^.

## Results and discussion

### Growth analysis and attributes

The observed improvements may be attributed to the role of humic acid in enhancing nutrient availability, root development, and physiological processes, rather than acting as a direct nutrient source. Increasing the HA concentration from 10 mLL^− 1^ to 20 mLL^− 1^ generally resulted in higher plant height, number of branches, pods per plant, fresh weight, and 100-seed weight (Table 3). The superior growth observed at HA 20 mL L^− 1^ may be explained by the stimulatory effect of humic substances on cell division and elongation, as well as their role in enhancing nutrient uptake and hormonal activity, particularly auxin-like effects. This could lead to increased vegetative growth, including plant height and branching. Similar findings have been reported in legume crops, where humic acid improved vegetative growth through enhanced physiological activity and nutrient assimilation^20,21^. Significant differences among genotypes were observed for all growth parameters. VC3896 showed the highest values for plant height, number of pods, and fresh weight, followed by King and Kawmy1, whereas AVM125 exhibited the lowest growth performance. These differences reflect genotypic variability in nutrient uptake efficiency and responsiveness to biostimulants. The interaction between genotype and HA treatment was significant, with King + HA 20 mLL^− 1^ showing the highest increase in plant height, branch number, and pods per plant, whereas Kawmy1 + HA 20 mLL^− 1^ recorded the highest 100-seed weight (4.94 g). These results suggest genotype-specific responses to HA, as reported in mungbean and other legumes^22–24^. The number of pods per plant and fresh weight were strongly correlated with seed yield, indicating that these traits can serve as reliable predictors of productivity (*r* = 0.97 for pods vs. fresh weight). The observed improvements in growth can be attributed to the role of humic substances in enhancing root development, nutrient availability, and membrane permeability, which collectively improve nutrient uptake and assimilation. Similar findings were reported by^25,26^ who demonstrated that humic acids stimulate root elongation and increase nutrient acquisition efficiency. In addition, HA has been shown to promote microbial activity in the rhizosphere, thereby improving soil fertility and plant growth.

### Yield and its components

This could be explained by Crop yield is a complex quantitative trait and the final product and vital goal of the farmer^27^. The yield of mungbean is determined by several yield component variables including pod number per plant, seed number per pod, 100-seed weight, and harvest index. The number of pods per plant is a key factor determining crop yield and higher yields are solely related to its multitude of pods bearing branches that produce more pods and number of seeds per pod^28^. Foliar application of humic acid increased seed, straw, biological and protein yields across all tested genotypes (Table 4). The 20 mLL^-1^ HA treatment produced the highest values for most parameters, except harvest index, which peaked at HA 10 mLL^-1^ in King (47.41%) King × HA 10 mLL^-1^ recorded the highest seed yield (3.85 ton ha^-1^ and protein yield (0.24 ton ha^-1^, whereas Kawmy-1 × HA 20 mLL^-1^ achieved the highest straw (6.38 ton ha^-1^ and biological yield (9.75 ton ha^-1^ This improvement may be attributed to the role of humic acid in enhancing nutrient availability, particularly nitrogen, phosphorus, and potassium, which are essential for photosynthesis and biomass accumulation. In addition, humic substances are known to stimulate root growth and microbial activity, thereby improving nutrient uptake efficiency. Humic substances are important components of organic matter, accounting for 60 to 70% of the total organic matter .The results of the study are shown in Table 4. Genotypic variation played a crucial role in determining yield performance indicated that King genotype recorded the highest value of seed, protein yield and harvest index (3.70, 0.23 ton ha^-1^ and 47.37%). But VC 3896 genotype gives the highest value of straw and biological yield (6.02 and 9.31 ton ha^-1^. However, VC1354 give the lowest value of all studied parameters. These results are identical to those obtained by some researchers they stated that the character of mungbean genotypes i.e. seed, biological and straw yield affected significantly^22–25^. The results in Table 4 also showed that the interaction between genotype of King and (HA 10 mLL^-1^ gave a significant increase on the seed, protein yield (3.85 and 0.24 ton ha^-1^. But harvest index (%) give the highest value with King with (control) by 49.93%. Meanwhile Kawmy^1^ + HA 20 mLL^-1^ recorded that highest value straw and biological yield under newly reclaimed soil conditions. (6.38 and 9.73 ton ha^-1^. These acids play an important role in plant growth by facilitating the absorption of essential nutrients, making them more available to plants. These results are confirmed by^29^ she found that using humic acid affected significantly on seed yield in wheat. Furthermore, HA application can lead to a reduction in the reliance on chemical fertilizers, thereby mitigating environmental pollution, and the associated lower consumption contributes to cost-effectiveness^30^. The observed variability suggests that selecting responsive genotypes and optimizing HA concentration can maximize productivity under newly reclaimed soils.

**Table 3:** Effect of foliar-applied humic acid on growth traits of mungbean genotypes.

Treatment		Plant height (cm)	Fresh weight of plant (g)	No of branches /plant	No of pods/plant	Weight of 100 seed (g)
Genotypes	Foliar spray(HA)					
Kawmy^1^	Control	51.67	73.90	3.55	41.32	4.43
	HA 10 mL L^-1^	61.83	89.86	4.23	48.19	5.63
	HA 20 mL L^-1^	75.53	106.76	4.82	50.92	4.94
King	Control	64.73	83.60	3.21	46.01	4.65
	HA 10 mL L^-1^	74.88	96.56	4.30	53.83	4.57
	HA 20	81.06	126.29	5.48	59.92	4.75
AVM125	Control mL L^-1^	46.51	63.89	1.75	35.23	3.65
	HA 10 mL L^-1^	52.36	80.09	2.75	38.88	3.94
	HA 20 mL L^-1^	62.34	97.06	3.75	48.69	4.15
VC1354	Control	44.29	62.16	2.25	34.76	4.29
	HA 10 mL L^-1^	48.72	75.11	3.25	39.50	4.54
	HA 20 mL L^-1^	65.17	94.74	4.25	48.92	4.87
VC3896	Control	65.63	94.88	2.75	50.26	4.65
	HA 10 mL L^-1^	76.08	106.48	3.75	52.91	4.47
	HA 20 mL L^-1^	80.87	124.60	5.25	59.95	4.81
Mean of genotype	Kawmy^1^	63.01	90.17	4.2	46.81	5.00
	King	73.56	102.15	4.33	53.25	4.65
	AVM125	53.74	80.35	2.75	40.93	3.91
	VC1354	52.72	77.34	3.25	41.06	4.56
	VC3896	74.19	108.65	3.91	54.37	4.64
Mean of foliar spray	Control	54.57	75.68	2.70	41.52	4.33
	HA 10 mL L^-1^	62.78	89.62	3.65	46.66	4.63
	HA 20 mL L^-1^	72.99	109.89	4.71	53.68	4.70
L.S.D 0.05	Genotypes	0.87	1.34	0.32	0.67	0.30
	Foliar spray	0.65	0.94	0.26	0.32	0.18
	Interaction	1.42	2.10	0.58	0.71	0.82

**Table 4:** Effect of foliar-applied humic acid on yield traits of mungbean genotypes.

Treatment		Seed yield (ton ha^-1^)	Straw yield (ton ha^-1^)	Biological yield (ton ha^-1^)	Protein yield (ton ha^-1^)	Harvest index (%)
Genotypes	Foliar spray (HA)					
Kawmy^1^	Control	3.24	4.14	7.38	0.19	43.95
	HA 10 mLL^-1^	3.32	5.10	8.42	0.20	39.48
	HA 20 mLL^-1^	3.37	6.38	9.75	0.20	34.53
King	Control	3.49	3.51	7.00	0.21	49.93
	HA 10 mLL^-1^	3.85	4.27	8.13	0.24	47.41
	HA 20 mLL^-1^	3.78	4.65	8.43	0.23	44.80
AVM125	Control	3.11	4.34	7.46	0.19	41.80
	HA 10 mLL^-1^	3.23	4.79	8.02	0.20	40.27
	HA 20 mLL^-1^	3.16	4.42	7.58	0.20	41.65
VC1354	Control	2.69	4.88	7.58	0.17	35.49
	HA 10 mLL^-1^	2.75	5.28	8.03	0.13	34.21
	HA 20 mLL^-1^	2.86	5.12	7.98	0.13	35.89
VC3896	Control	3.00	5.94	8.95	0.13	33.56
	HA 10 mLL^-1^	3.38	6.43	9.82	0.17	34.37
	HA 20 mLL^-1^	3.47	5.70	9.17	0.19	37.80
Mean of genotype	Kawmy^1^	3.30	5.20	8.51	0.20	39.32
	King	3.70	4.14	7.85	0.23	47.37
	AVM125	3.16	4.51	7.68	0.20	41.26
	VC1354	2.70	5.09	7.86	0.14	35.19
	VC3896	3.20	6.02	9.31	0.17	35.27
Mean of foliar spray	Control	3.10	4.56	7.67	0.18	40.96
	HA 10 mLL^-1^	3.30	5.17	8.48	0.19	39.16
	HA 20 mLL^-1^	3.32	5.25	8.58	0.19	38.93
L.S.D 0.05	Genotypes	0.07	0.20	0.22	0.01	1.17
	Foliar spray	0.65	0.19	0.18	0.01	1.14
	Interaction	0.15	0.42	0.41	0.01	2.56

### Nutritional composition of mungbean seeds

**Fig. 1:**
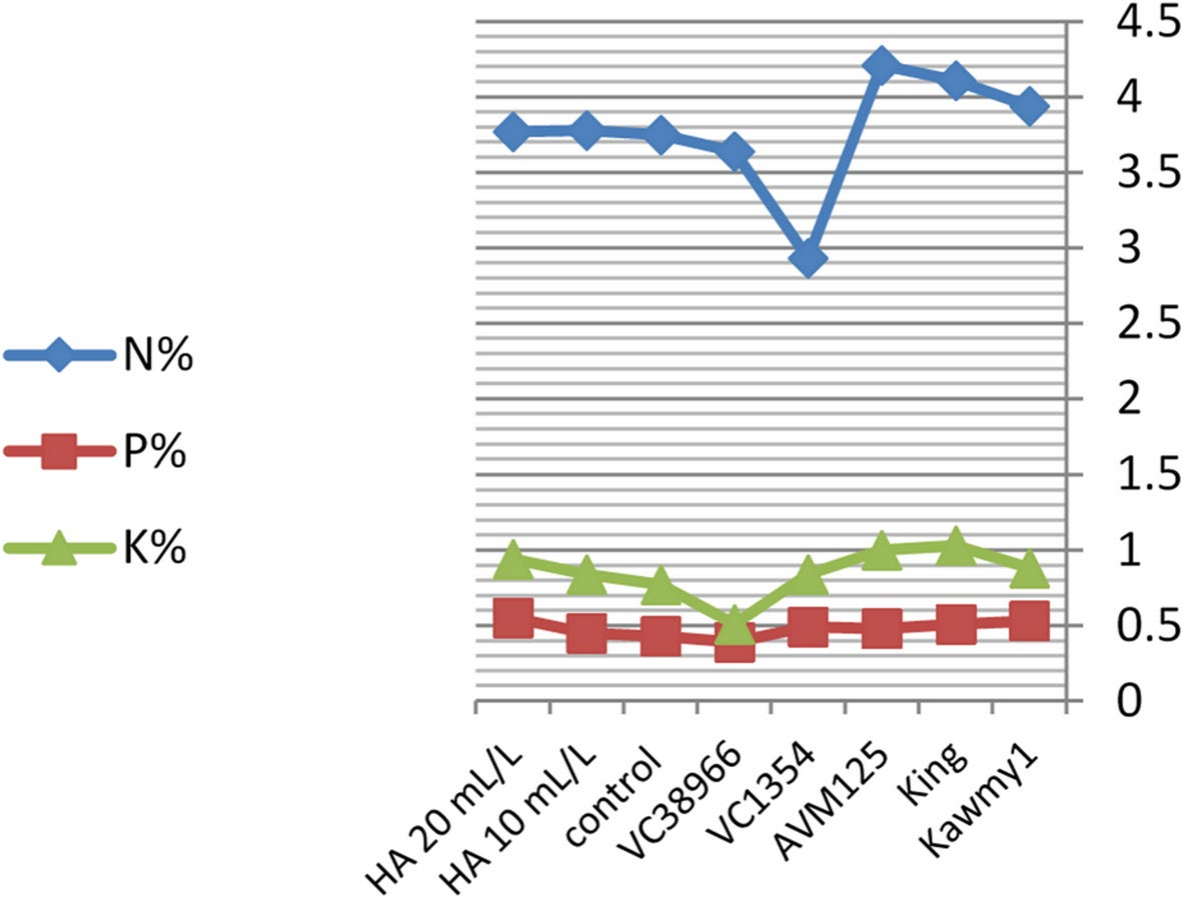
Effect of foliar-applied humic acid on nutrient content (N, P, and K) of mungbean genotypes.

**Fig. 2:**
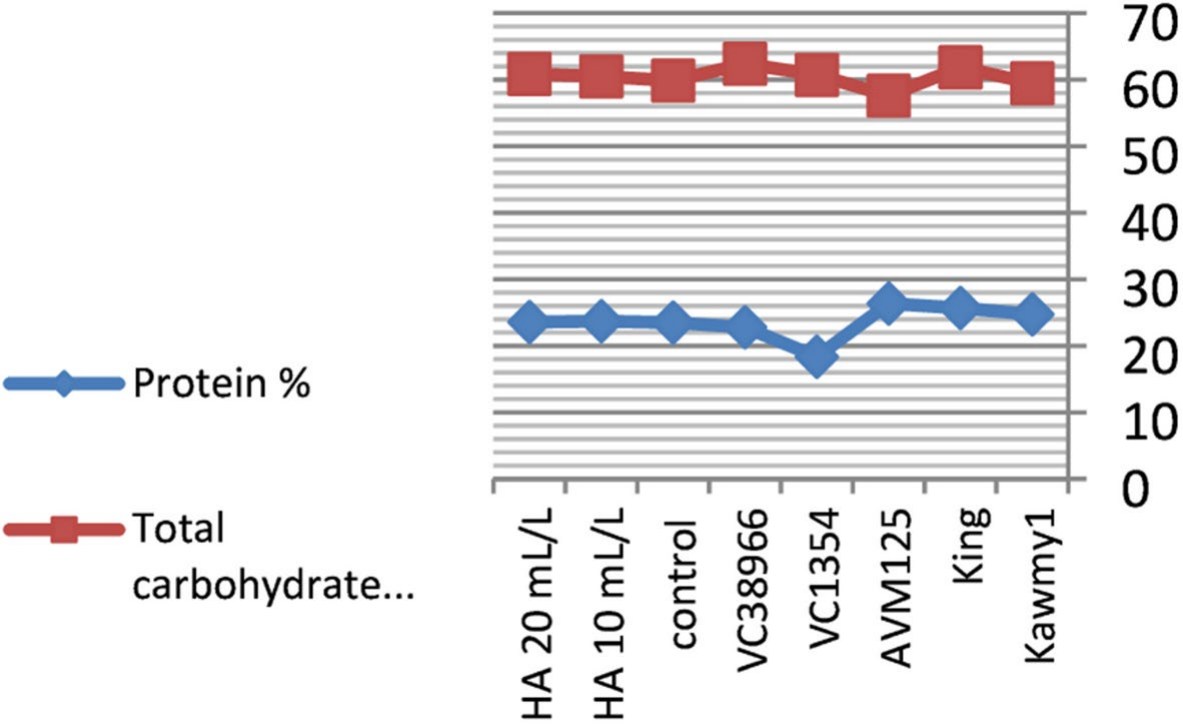
Effect of foliar -applied humic acid on Protein% and T. carbohydrate% on some mungbean genotypes.

**Fig. 3:**
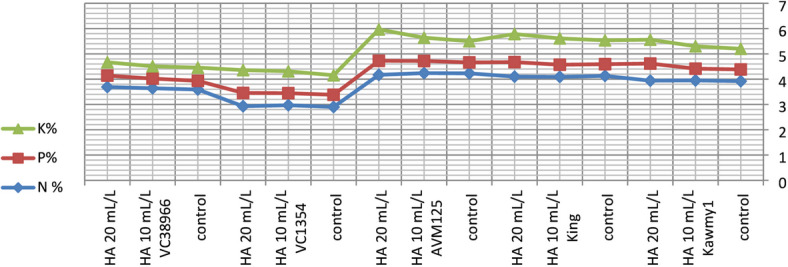
Interaction effects of humic acid foliar application and genotype on chemical composition of mungbean.

**Fig. 4:**
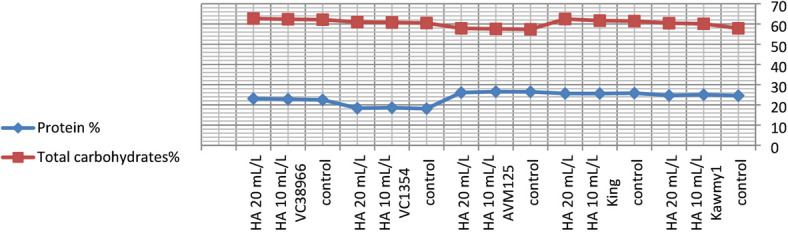
Interaction effects of humic acid foliarapplication and genotype on chemical composition of mungbean.

Foliar application of humic acid significantly enhanced nutrient accumulation and seed biochemical composition of mungbean cultivated under the conditions of this study^7^ (Figs.  1, 2, 3 and 4). The application of HA at 20 mL L^–1^ resulted in the highest potassium and phosphorus contents (0.94% and 0.55%, respectively), accompanied by increased carbohydrate accumulation (60.87%), indicating improved nutrient uptake and photosynthetic assimilate production compared with untreated plants. Maximum protein content was obtained under HA at 10 mL L^–1^ (23.71%), whereas a slight decline was observed at the higher rate (23.57%), suggesting that moderate humic acid levels optimize nitrogen assimilation and protein synthesis, while higher concentrations mainly stimulate carbohydrate formation. The relatively stable nitrogen percentage across treatments indicates improved nitrogen use efficiency rather than increased nitrogen accumulation^31,32^. Marked genotypic differences were detected for all nutritional traits. The genotype *King* exhibited high nitrogen (4.11%) and carbohydrate contents (61.86%), reflecting superior nutrient acquisition efficiency, whereas *AVM125* recorded the highest protein content (26.36%), indicating efficient nitrogen utilization^33^. In contrast, *VC1354* showed the lowest nitrogen (2.93%) and protein content (18.36%), suggesting limited responsiveness under reclaimed soil conditions. Genotype × humic acid interaction revealed differential responses, where *AVM125* achieved maximum protein content (26.53%) under HA at 10 mL L^–1^, while *Kawmy*^1^ attained the highest phosphorus (0.677%) and potassium (1.11%) contents under HA at 20 mL L^–1^. Overall, humic acid application improved seed nutritional quality through enhanced nutrient efficiency and balanced carbon–nitrogen metabolism, with genotype selection playing a key role in maximizing mungbean performance under nutrient-limited soils.

### Chemical composition of amino acid

**Fig. 5:**
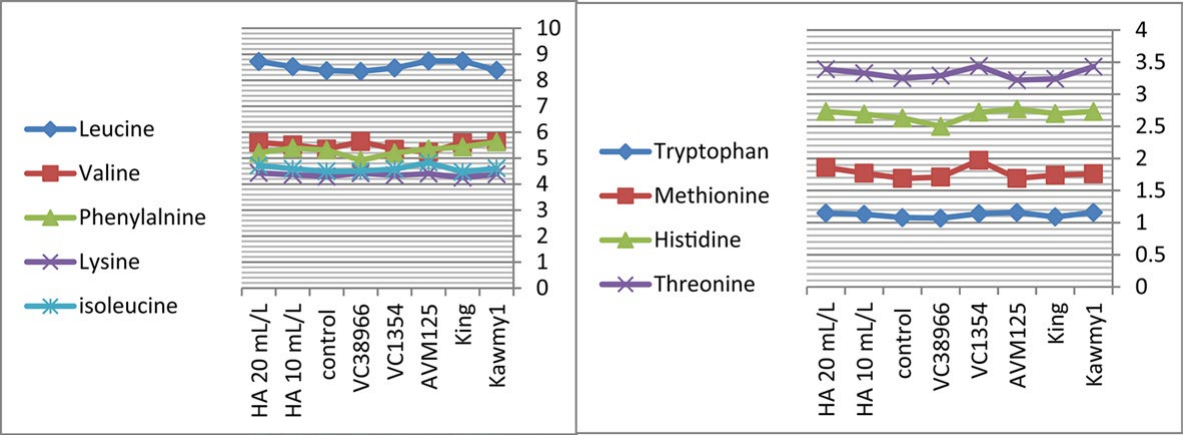
Effect of foliar-applied humic acid on essential amino acids of mungbean genotypes.

**Fig. 6:**
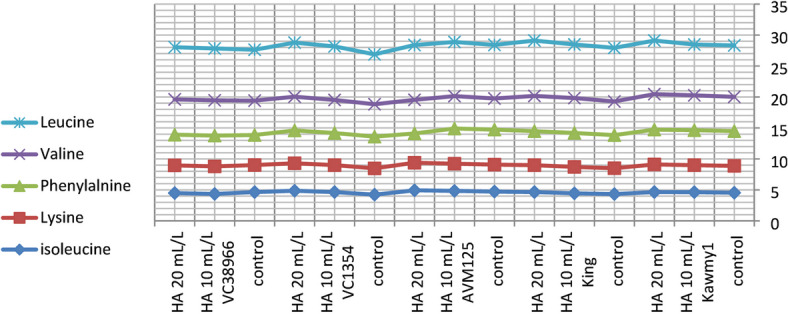
Interaction effect of foliar-applied humic acid on essential amino acids of mungbean genotypes.

**Fig. 7:**
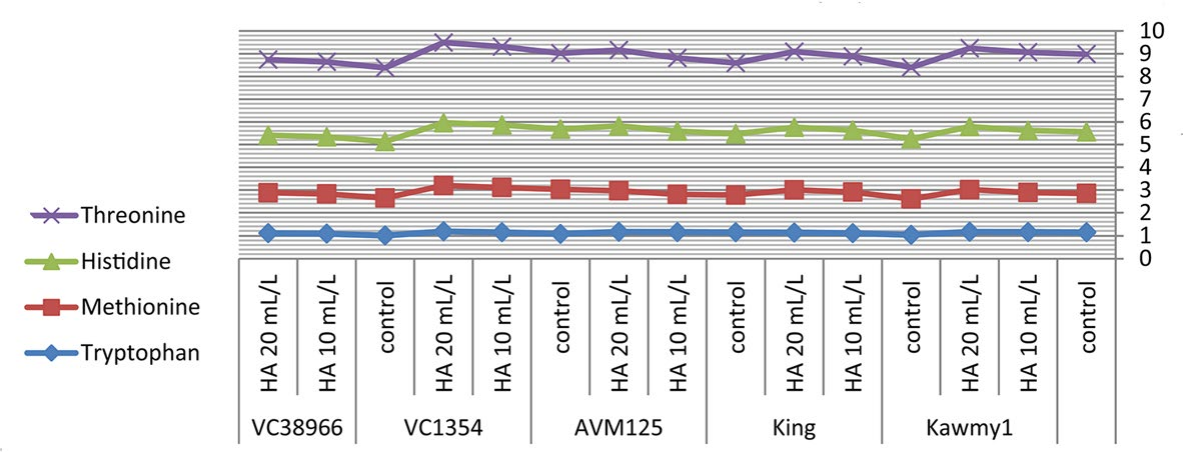
Interaction effect of foliar-applied humic acid on essential amino acids of mungbean genotypes.

Amino acid composition represents a key indicator of seed nutritional quality, as amino acids constitute the fundamental structural units of proteins and play essential roles in plant metabolism and stress adaptation. In addition to their structural function, amino acids participate in enzymatic regulation, nutrient transport, and osmotic adjustment processes that contribute to plant growth and productivity under adverse environmental conditions. Therefore, evaluating essential amino acid profiles provides important insight into the physiological responses of mungbean to humic acid application under newly reclaimed soils. Figure 5 HA at 20 mL L^-1^ produced the highest potassium and phosphorus contents (0.94% and 0.55%, respectively) and increased total carbohydrate accumulation (60.87%), reflecting improved nutrient uptake and photosynthetic efficiency. Maximum protein content occurred under HA at 10 mL L^-1^ (23.71%), slightly decreasing at 20 mL L^-1^ (23.57%), indicating that moderate humic acid optimizes nitrogen assimilation and protein biosynthesis, while higher concentrations favor carbohydrate formation. Nitrogen percentages were relatively stable, suggesting enhanced nitrogen utilization efficiency rather than total accumulation^10^. The increase in essential amino acids such as lysine (4.29), methionine (1.86), and leucine (8.72) under HA application suggests an enhancement in protein biosynthesis pathways. This may be linked to improved nitrogen assimilation and enzyme activity associated with amino acid metabolism. The higher response observed at 20 mL L^-1^ indicates that humic acid may influence metabolic pathways beyond basic nutrition, possibly through its role as a biostimulant affecting gene expression and enzymatic regulation. While HA at 10 mL L^-1^ maximized phenylalanine (5.39), highlighting the selective regulation of amino acid synthesis pathways. Genotypic variation was also evident, with some genotypes showing higher amino acid accumulation than others. This indicates that genetic factors play a crucial role in determining metabolic response to biostimulants. *King* showed high nitrogen (4.11%) and carbohydrate contents (61.86%), reflecting superior nutrient acquisition; *AVM125* recorded the highest protein (26.36%) and amino acids including leucine (8.74), isoleucine (4.83), histidine (2.77), and tryptophan (1.16); *Kawmy1* excelled in lysine (4.34) and phenylalanine (5.62); *VC1354* performed best in methionine (1.70) and threonine (3.44), whereas *VC38966* generally displayed lower amino acid levels, indicating weaker metabolic responsiveness. The results in Figs. 6 and 7. The significant interaction between genotype and HA treatment highlights the importance of selecting responsive genotypes to maximize seed nutritional quality. Showing that HA at 20 mL L^-1^ synergistically enhanced lysine, methionine, leucine, and valine in high-performing genotypes, while *VC38966* maintained the lowest levels of several amino acids. Overall, these findings demonstrate that seed nutritional quality is governed by both genetic background and humic acid application, which collectively enhance nutrient uptake, protein synthesis, and amino acid metabolism, ultimately improving seed biochemical composition and overall productivity under nutrient-limited reclaimed soils^34^.

**Fig. 8:**
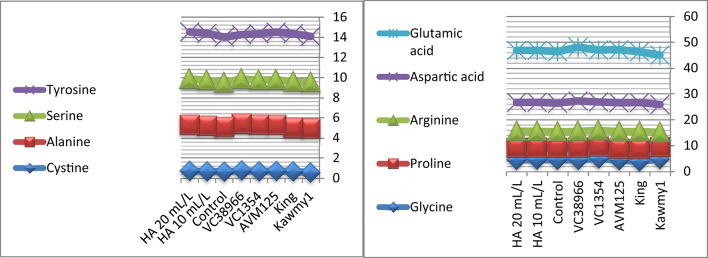
Effect of foliar-applied humic acid on non- essential amino acids of mungbean genotypes.

**Fig. 9:**
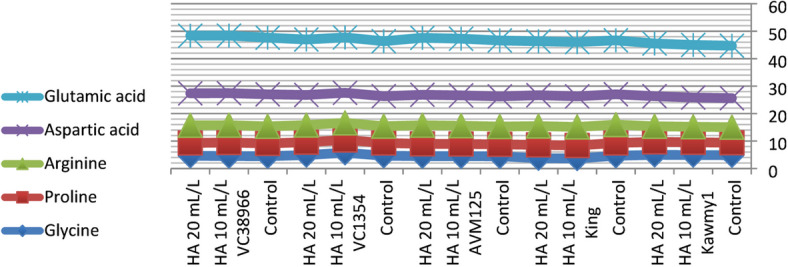
Interaction effect of foliar-applied humic acid on non -essential amino acids of mungbean genotype.

**Fig. 10:**
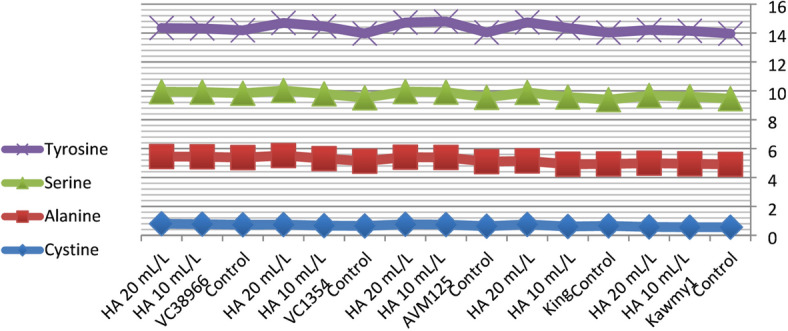
Interaction Effect of foliar-applied humic acid on non- essential amino acids of mungbean genotypes.

Foliar application of humic acid significantly influenced the accumulation of non-essential amino acids in mungbean seeds across different genotypes (Figs. 8, 9 and 10). The control treatment displayed relatively balanced amino acid concentrations, whereas HA application at 10 and 20 mL L^-1^ generally enhanced glutamic acid, aspartic acid, and arginine levels, highlighting the stimulatory effect of humic acid on nitrogen metabolism and protein-related intermediates^20^. Genotypic variation was evident: *VC38966* exhibited the highest glutamic acid content (20.99), while *Kawmy1* had the lowest (19.10). Aspartic acid ranged from 10.58 in *Kawmy1* to higher values in other genotypes, and arginine showed its lowest concentrations in *Kawmy1* (5.55–5.65) versus a maximum in *King* (6.85), with *King* and *VC38966* demonstrating the most pronounced increases. Proline levels varied minimally (4.45–4.74), with the highest value in *King*, indicating relative stability across treatments. Glycine exhibited greater variability among genotypes, reaching 5.75 in *VC1354* and the lowest value of 3.65 in *King*, suggesting stronger genetic influence than treatment effect. Overall, the interaction between genotype and humic acid application confirmed that increasing HA concentration from 10 to 20 mL L^-1^ selectively enhanced glutamic acid, aspartic acid, and arginine, while proline remained stable and glycine trends were genotype-dependent. These results demonstrate that humic acid acts as a metabolic biostimulant, enhancing non-essential amino acid synthesis and nitrogen assimilation in a genotype-specific manner, ultimately contributing to improved seed biochemical composition under nutrient-limited reclaimed soils^35^.

### Correlation

Correlation analysis revealed strong positive relationships between growth traits and yield components (Table 5). The number of pods per plant and fresh weight were highly correlated (*r* = 0.97), emphasizing their predictive value for yield performance. Protein yield correlated positively with seed yield (*r* = 0.85), while straw yield showed a slight negative association with seed yield (*r* = − 0.19), indicating potential trade-offs between vegetative biomass and reproductive output. Chemical correlations (Table 6) revealed strong positive associations between K% and P% (*r* = 0.65), and K% and N% (*r* = 0.37) Overall, correlation analysis confirms that improving vegetative growth, nutrient uptake and physiological efficiency through HA application can lead to enhanced yield and seed quality in mungbean under reclaimed soil conditions.

### Limitations of the study

Despite the promising results obtained in this study, some limitations should be considered. The experiments were conducted at a single location (Nubaria) over two growing seasons, which may limit the generalization of the findings to other environmental conditions. Additionally, only two concentrations of humic acid were evaluated, and a wider range of application rates could provide a more comprehensive understanding of dose–response relationships. Furthermore, economic feasibility and long-term effects on soil properties were not assessed. Therefore, further multi-location and long-term studies are recommended to validate these findings under diverse agro-ecological conditions.

## Conclusion

The present study demonstrated that foliar application of humic acid (HA) significantly improved growth performance, yield components, and seed quality of mungbean genotypes under newly reclaimed soil conditions. The application of HA at 20 mL L^-1^ enhanced vegetative growth traits, while HA at 10 mL L^-1^ was more effective in optimizing protein accumulation. Clear genotypic variation was observed, with King and VC3896 showing superior productivity and yield traits, whereas AVM125 exhibited comparatively lower growth performance under the tested conditions. The interaction between genotype and HA level played a significant role in determining yield and quality attributes, indicating that genotype selection is crucial for maximizing the benefits of HA application. Improvements in nutrient uptake (N, P, and K), carbohydrate accumulation, and amino acid composition suggest that humic acid contributes to enhanced physiological efficiency and seed nutritional value. Under the conditions of this study, foliar application of humic acid may represent a promising agronomic practice for improving mungbean productivity and seed quality in newly reclaimed soils. However, further research under diverse environmental conditions is required to confirm these findings and support wider agricultural application.

**Table 5:** Correlation coefficients between different mungbean characteristics.

Straw yield	Protein yield	HI %	Bio yield	Seed yield	100 seed	No of *P*/plant	No of B/plant	F.W (g)	Plant H(cm)	
										plant H (cm)
									0.9462596	F.W (g)
								0.8250258	0.7886773	No of B/plant
							0.8199792	0.9631343	0.9666063	No of P/plant
						0.4603599	0.5151303	0.3960513	0.4299811	100 wt.seed
					0.1596687	0.6640646	0.5730825	0.6009479	0.7028808	Seed yield
				0.2223052	0.2998683	0.5723726	0.4381583	0.6410883	0.6286681	Bio yield
			-0.6058509	0.6337896	-0.1042914	0.0809286	0.0983874	-0.0266934	0.0801452	HI %
		0.72299	-0.0974059	0.8067784	-0.0319818	0.2739552	0.3299547	0.2635399	0.3317142	Protein Y
	-0.4173544	-0.8627729	0.9227902	-0.1704972	0.2406572	0.3158729	0.2161671	0.4101833	0.3573878	Straw yield

**Table 6:** Correlation coefficients between different chemical analyses of seed in mungbean.

K%	*P*%	*N* %	Total carbohydrates%	Protein %	
					Protein %
				-0.3360162	Total carbohydrates%
			-0.3351404	0.9984822	N %
		0.1179181	-0.1334691	0.1188928	P%
	0.6471811	0.3676161	-0.3838969	0.3681605	K%

## Data Availability

The datasets used and/or analyzed during the current study available from the corresponding author on reasonable request.
